# Management of the Placenta

**Published:** 1871-01

**Authors:** 


### MANAGEMENT OF THE PLACENTA.

After the expulsion of the child, and the division of the cord, the patient still in the usual position, the left hand should be passed over the abdomen to ascertain the condition of the uterus. If contracted, and the placenta extruded from the cavity, it only remains to extract it with the right hand from the vagina. If, however, the uterus be flaccid, and increasing in size, commence and keep up pressure upon that organ until the progressive shrinking of its bulk induces the expulsion of the contained coagula, and the close compressure of the placental mass. Then wait some five or ten minutes. After this time, grasp the uterus, and by external pressure cause it to drive the placenta into the vagina, whence it may be withdrawn by seizing an edge with the fingers. Do not trust the umbilical cord.

The advantages thus secured are the certainty as to the actual state of the uterus; the control obtained over the quantity of blood poured out during the process of detachment of the plancenta; the ultimate attainment of complete contraction; the almost complete emptying of the uterine cavity from coagula, etc.; the much increased certainty that concealed or post-partum hemorrhage will not occur; the absence of hour-glass contraction, or undue detention of the placenta.—*British Medical Journal.*

